# Transcriptional Profile of *Bacillus subtilis sigF*-Mutant during Vegetative Growth

**DOI:** 10.1371/journal.pone.0141553

**Published:** 2015-10-27

**Authors:** Wout Overkamp, Oscar P. Kuipers

**Affiliations:** 1 Department of Molecular Genetics, Groningen Biomolecular Sciences and Biotechnology Institute, University of Groningen, Groningen, The Netherlands; 2 Kluyver Centre for Genomics of Industrial Fermentation, Delft, The Netherlands; ContraFect Corporation, UNITED STATES

## Abstract

Sigma factor F is the first forespore specific transcription factor in *Bacillus subtilis* and controls genes required for the early stages of prespore development. The role of *sigF* is well studied under conditions that induce sporulation. Here, the impact of *sigF* disruption on the transcriptome of exponentially growing cultures is studied by micro-array analysis. Under these conditions that typically don’t induce sporulation, the transcriptome showed minor signs of sporulation initiation. The number of genes differentially expressed and the magnitude of expression were, as expected, quite small in comparison with sporulation conditions. The genes mildly down-regulated were mostly involved in anabolism and the genes mildly up-regulated, in particular fatty acid degradation genes, were mostly involved in catabolism. This is probably related to the arrest at sporulation stage II occurring in the *sigF* mutant, because continuation of growth from the formed disporic sporangia may require additional energy. The obtained knowledge is relevant for various experiments, such as industrial fermentation, prolonged experimental evolution or zero-growth studies, where sporulation is an undesirable trait that should be avoided, e.g by a *sigF* mutation.

## Introduction

During conditions of nutrient starvation *Bacillus subtilis* is able to form dormant endospores, which can survive exposure to high temperatures, ultraviolet radiation, chemical solvents and other extreme conditions [1]. Endospore formation happens through asymmetric cell division, followed by engulfment of the smaller forespore by the larger mother cell. Development of the forespore into a spore is facilitated by the mother cell, which upon completion lyses and releases the spore (For reviews see [2–4].

The differentiation process is initiated by DNA-binding protein Spo0A, which in turn triggers programs of gene expression specific for the forespore and mother cell. Spo0A is a crucial transcriptional regulator with several hundred genes directly [5] and indirectly under its control [6]. It is activated by phosphorylation, which is governed by the phosphorelay [7]. The phosphorelay integrates environmental and intracellular signals to interpret whether a cell should initiate sporulation or not, and thus functions as a complex regulatory system [8].

The initial stages of forespore and mother cell development are governed by RNA polymerase sigma factors σ^F^ and σ^E^, respectively. Their expression is controlled by Spo0A~P [2,9,10] and both are produced before formation of the asymmetric septum and remain inactive until completion of the septum [11]. The first forespore specific transcription factor σ^F^ controls genes required for the early stages of prespore development and is required for activation of σ^E^ [2,9,10]. Additionally, it directs transcription of the gene encoding σ^G^, which replaces σ^F^ in later stages of prespore development. The successive activation of specific sigma-factors ensures that the forespore and mother-cell-specific programs of gene expression are kept in pace with the morphogenesis. The gene *sigF* is part of the three-cistron *spoIIA* operon and is also named *spoIIAC* [12]. The other members *spoIIAA* and *spoIIAB* are involved in regulation of σ^F^ activity [9,13–15].

During circumstances where sporulation is undesirable, such as with industrial fermentations, evolution experiments and particular chemostat experiments, the use of sporulation-deficient strains can be a solution. In *Bacillus subtilis* a disruption of *sigF* prevents continuation of sporulation at stage II [16,17]. Therefore, it is relevant to assess the effect of a *sigF* mutation on the general metabolism and regulatory circuitry during vegetative growth.

The phenotype and genotype of *B*. *subtilis* carrying a disruption in *sigF* have previously been characterized under sporulation-inducing conditions [6,18–20]. Cells lacking σF are unable to complete differentiation into a forespore and instead form an additional polar septum at the opposite cell pole [17,21]. When excess of nutrients is available, these disporic sporangias are able to reinitiate growth from both polar compartments [18].

Transcriptome studies of *sigF* mutant strains that were grown under sporulation conditions and studies where *sigF* was artificially induced, revealed SigF-dependent genes and the essential role of *sigF* in the sporulation process [6,19,20]. Although the *sigF* mutation will not affect micro-array transcript ratios in typical experiments that analyze the response of an isogenic strain to varying conditions, it is of interest to assess the impact of the *sigF* mutation against a wild-type background under vegetative conditions.

Here we study the impact of the *sigF* disruption on the transcriptome of exponentially growing cultures. The transcriptome of a *sigF* mutant is compared to that of the wild-type strain using micro-array analysis. We find that under these non-sporulation-inducing conditions the transcriptome showed minor signs of sporulation initiation, and that the number of differentially expressed genes and the magnitude of expression were quite small in comparison with those under sporulation conditions. The down-regulated genes are mostly involved in anabolism and the up-regulated genes are mostly involved in catabolism, in particular fatty acid degradation genes. This probably reflects the arrest at sporulation stage II of the *sigF* mutant, redistributing energy over the polar compartments to resume normal growth.

## Material and Methods

### Bacterial strains, plasmids, media and growth conditions

Bacterial strains and plasmids used in this study are listed in Table 1. *Bacillus subtilis* strain *sigF*::*spc* was obtained by a double recombination event of plasmid pUC18sigF::spc into the chromosomal *sigF* region of *B*. *subtilis* 168 *trpC2*. Correct integration into the chromosome was checked by PCR and DNA sequencing.

**Table 1:** Bacterial strains and plasmids used in this study^a^.

Strains and plasmids	Relevant properties	Source or reference
*E*. *coli* DH5α	F-, *araD*139, Δ(*ara-leu*)7696, Δ(*lac*)X74, *galU*, *galK*, *hsdR2*, *mcrA*, *mcrB1*, *rspL*	Laboratory stock
*B*. *subtilis*		
168	*trpC2*	[22]
* sigF*::*spc*	168, *sigF*::*spc*, Sp^r^	This study
Plasmids		
pUC18	*lacZ*, Amp^r^	[23]
pUC18sigF	pUC18 derivative carrying *sigF*. Amp^r^	This study
pUC18sigF::spc	pUC18 derivative carrying *sigF* with *spc* cassette. Amp^r^, Sp^r^	This study
pDG1727	Sp^r^	[24]

^a^ Abbreviations: Amp^r^, ampicillin resistance; Sp^r^, spectinomycin resistance

To construct plasmid pUC18sigF::spc, carrying a spectinomycin resistance cassette flanked by the 5`and 3`ends of *sigF*, a PCR with the primers sigF-Fw and sigF-R was performed to amplify the *sigF* gene region, using chromosomal DNA of *B*. *subtilis* 168 as template. The PCR fragment and pUC18 were digested with the restriction enzyme HincII and ligated into the corresponding site in pUC18, yielding the plasmid pUC18sigF. Subsequently, to exchange the majority of the *sigF* gene with a spectinomycin resistance cassette, leaving only the flanking regions, a round PCR was performed on pUC18sigF using the primers sigF-SalI-Fw and sigF-XhoI-R. The PCR fragment and the plasmid pDG1727, harbouring the spectinomycin resistance cassette, were both digested with XhoI and SalI. A ligation was then performed on the PCR fragment and the gel-isolated spectinomycin resistance cassette to yield the plasmid pUC18sigF::spc. Oligonucleotides used in this study are listed in Table 2.

**Table 2:** Oligonucleotides used in this study.

Primers	Sequence (5`to 3`)	Description; position
sigF-Fw	GCTTGAATTCGATGGCAAGACACGATCC	on *spoVAA (*downstream of *sigF)*
sigF-R	GTCGCTGCAGGAACAATCTGAACAGCAGGCACTC	on *spoIIAB* (upstream of *sigF*)
sigF-SalI-Fw	GCACGTCGACAACCTCCACATCCATAAC	*SalI*; on 5’ of *sigF*
sigF-XhoI-R	GCTTCTCGAGGCCATACGGATGGCTAGTCTG	*XhoI*; on 3’ of *sigF*

Restriction sites are underlined.

*B*. *subtilis* was grown at 37°C on Trypton Yeast-extract (TY) medium (Sambrook et al. 1989) solidified with 1.5% (wt/vol) agar, or in TY or Difco Sporulation Medium (DSM) [25] with shaking at 200 rpm (see below). *Escherichia coli* DH5α was used as host for cloning and grown in TY medium at 37°C with shaking or on TY medium solidified with 1.5% (wt/vol) agar. When required, the growth media were supplemented with the following antibiotics: 100 μg ml^−1^ ampicillin (amp) or 150 μg ml^−1^ erythromycin (em) for *E*. *coli*, 100 μg ml^−1^ spectinomycin (spc) for *B*. *subtilis*.

### Recombinant DNA techniques and oligonucleotides

DNA purification, restriction, ligation, agarose gel electrophoresis and transformation of *E*. *coli* was performed as described before [26]. Oligonucleotides were purchased from Biolegio (Nijmegen, the Netherlands). Enzymes were purchased from New England Biolabs (Ipswich, MA, USA) and Fermentas (Vilnius, Lithuania) and used as prescribed by the manufacturer. *B*. *subtilis* was transformed as described previously [27].

### Determination of sporulation frequencies

For sporulation conditions *B*. *subtilis* 168 and *sigF*::*spc* were inoculated into DSM and incubated at 37°C with shaking at 200 rpm for 24 h. For vegetative conditions *B*. *subtilis* 168 and *sigF*::*spc* were inoculated into TY and grown to OD_600_ of 1.5. Cultures were diluted 1/10 into 1.5 ml of spizizen salts [28] and 100 μl chloroform was added. The cell suspensions were mixed and incubated for 30 min at 80°C. Serial dilutions of treated and untreated cultures were plated on TY solidified with 1.5% (wt/vol) agar for CFU counting.

### Growth and preparation of cells for RNA isolation

Cultures were grown in absence of antibiotics in 250-ml Erlenmeyer flasks by inoculation of 25 ml medium with cells at OD_600_ 0.05. For vegetative conditions, cells were grown in TY medium, subsequently diluted and regrown to synchronize cultures and to minimize carryover of sporulating cells, and finally samples for RNA isolation were taken at OD_600_ 1.5. For sporulation conditions cells were grown in DSM medium and samples for RNA isolation were taken 2 hours after the transition point between the exponential and stationary growth phase. Cell pellets were immediately frozen in liquid nitrogen and stored at -80°C until RNA isolation.

### DNA microarray analysis

Design and production of DNA microarrays was done according to standardized lab protocols described previously [29].

For RNA isolation, cell culture samples were quickly centrifuged for 2 min at 6,000 × g, and frozen in liquid nitrogen. Cells were broken using 500 mg of glass beads, 500 μl of phenol-chloroform, 30 μl of 3 M sodium acetate, and 15 μl of 20% sodium dodecyl sulfate. RNAs were isolated using the High Pure RNA isolation kit (Roche, Mannheim, Germany) according to the manufacturer's instructions. After a quality check of the isolated RNA using an Agilent Bioanalyzer 2100 with RNA 6000 LabChips (Agilent Technologies, the Netherlands), 20 μg of total RNA was used for cDNA synthesis and incorporation of aminoallyl-dUTP using SuperscriptIII reverse transcriptase (Invitrogen, Life Technologies Europe BV, the Netherlands). Subsequently, the cDNA was labeled with Cy3- or Cy5-monoreactive dye (Amersham Biosciences) and hybridized to oligonucleotide microarrays as described before [29,30]. Slides were scanned using a confocal laser scanner (GenePix Autoloader AL4200, Molecular Devices Ltd., Sunnyvale, USA). Fluorescent signal intensity data were quantified using ArrayPro (Media Cybernetics Inc., Silver Spring, Md., USA) with a local corners background correction. The obtained expression levels were subjected to a t-test using CyberT software [31] after the values were processed and normalized (Lowess method) using microPreP [32]. For both vegetative- and sporulation conditions the experimental setup included two biological replicates with a dye swap for one of the replicates, resulting in three slides. In total six measurements per gene were performed, since two duplicate spots for all genes were present on each slide. Genes having a fold change higher then 2 and a Bayes p-value lower than 0.05 were considered to be expressed differentially. The gene lists selected with these criteria are presented in S1 and S2 Tables. The software package FIVA (Functional Information Viewer and Analyzer; [33] was used to identify overrepresented functional categories in differentially expressed genes. Sources used by this software include: metabolic pathways from Kyoto Encyclopedia of Genes and Genomes (KEGG; [34]), categories from Gene Ontology (GO; [35]) and Cluster of Orthologous Groups (COG; [36]) and regulons from Database of Transcriptional regulation in *Bacillus subtilis* (DBTBS; [37]). DNA microarray data are available in the Gene Expression Omnibus database (GEO; http://ncbi.nlm.nih/gov/geo/) under the accession number GSE64918.

## Results & Discussion

### Construction of *B*. *subtilis sigF* mutant strain

An asporogenous strain was constructed by replacing bp 2443565 to 2444182 of the *B*. *subtilis 168* chromosome [22] with a spectinomycin resistance cassette. This area corresponds to majority of the gene *sigF*. To confirm deficiency in spore formation, the *sigF* strain and wild-type strain were allowed to sporulate in Difco Sporulation Medium (DSM) and were analyzed for the presence of heat- and chloroform- resistant spores by counting Colony Forming Units (CFU). Sporulation frequency of the *sigF* mutant strain was determined to be equal or less than 0.0001%, compared to 14% of the wild-type strain. These numbers confirm that the *sigF* mutant strain is unable to form spores.

### Initiation of sporulation cascade hampered in *sigF* deletion strain

To confirm that sporulation genes were not transcribed in the *sigF* mutant strain, its transcriptome profile was compared with the wild-type strain under sporulation conditions. A total number of 958 genes, of which 758 down-regulated and 200 up-regulated, were differentially expressed in the *sigF* mutant strain versus the wild-type strain. The majority of the down-regulated genes have a function in -or related to- sporulation (S1 and S2 Tables), as is described in various other studies [6,19,20]. As expected, functional analysis with the FIVA software tool confirms that sporulation is the main functional group affected by the *sigF* mutation (S1 Fig).

Genes directly activated by SigF, such as *rsfA*, *sigG*, *gpr* and *spoIIQ*, as well as genes transcribed by other sigma factors later in the sporulation process are clearly expressed to a lesser extent in the *sigF* mutant strain. In contrast, for genes that are transcribed earlier in the sporulation cascade than *sigF*, such as *spoIIE*, *spoIIGA*, *sigE (spoIIGB)*, *and sigH (spo0H)*, *the transcript ratios* are equal between mutant and wild-type strain. The *sigF* mutation thus prevents completion of sporulation, but still allows for determination whether sporulation is initiated by monitoring of genes upstream of *sigF*.

### Transcriptional profile under non-sporulation conditions

The role of SigF in vegetative growth was examined by comparing the transcriptional profiles of *B*. *subtilis sigF*::*spc* cells with its isogenic wild-type strain. Samples for DNA-microarray analysis were collected in the mid-exponential phase. Previously it has been reported that 0.4% of the cells form spores during exponential growth in casein hydrolysate medium [38]. In contrast, the presence of matured spores in our exponentially growing culture of the wild-type strain is highly unlikely, since a sporulation CFU count of vegetative growing cultures showed that the sporulation frequency is equal or less than 0.001% at this time point for both wild-type and *sigF* mutant strain. Notably, such a sporulation assay requires chloroform treatment that kills everything except matured spores and even cells that have initiated but did not complete spore formation will not survive. Therefore, the fraction of the culture that has initiated the sporulation process might actually be larger than observed.

The *sigF* regulon is well-studied and previous studies have shown that artificial induction of *sigF* under exponential conditions results in a similar sporulation-specific transcriptome response as occurs during sporulation initiation triggered by environmental cues [19,20]. In our experiment, the majority of the differentially expressed genes under vegetative conditions are not known to be *sigF-*regulated or to be sporulation-specific, indicating a more general response.

A total of 117 genes were differentially expressed in the *sigF* mutant strain versus the wild-type strain (for a top list see Table 3). The number of down-regulated and up-regulated genes was 68 and 49, respectively (S3 and S4 Tables). 35 of these genes were also differentially expressed in the sporulation-condition, among which 8 are listed to be involved with sporulation [39,40]. Thus, the difference in transcript profiles between the mutant and wild-type strain is caused only to a small extent by regulation directly related to sporulation.

**Table 3:** Toplist of genes differentially expressed in *B*. *subtilis* 168 *sigF*::*spc* under vegetative conditions.

Gene/Operon	Product	Fold change
*yosX*	hypothetical protein	-4.33
*yjgC*	oxidoreductase	-3.61
*yjgD*	hypothetical protein	-3.38
*yotB*	metallo-dependent hydrolase	-3.36
*ysnF*	stress response protein	-3.17
*opuCA*	glycine betaine/carnitine/choline/choline sulfate ABC transporter ATP-binding protein	-2.91
*yknU*	ABC transporter ATP-binding protein	-2.88
*spoIIAB*	anti-sigma F factor	-2.87
*ygxB*	hypothetical protein	-2.83
*pbpX*	penicillin-binding endopeptidase X	-2.76
*yorO*	hypothetical protein	-2.70
*purH*	bifunctional phosphoribosylaminoimidazolecarboxamide formyltransferase/IMP cyclohydrolase	-2.70
*yotC*	hypothetical protein	-2.67
*yukC*	bacteriocin production protein	-2.67
*yflT*	heat stress induced protein	-2.58
*yhcW*	phosphoglycolate phosphatase	-2.49
*comGF*	DNA transport platform protein	-2.49
*leuC*	isopropylmalate isomerase large subunit	-2.44
*purN*	phosphoribosylglycinamide formyltransferase	-2.44
*yxeD*	hypothetical protein	11.84
*fadE*	acyl-CoA dehydrogenase	7.07
*fadA*	acetyl-CoA acetyltransferase	4.70
*fadN*	enoyl-CoA hydratase	4.07
*iolT*	myo-inositol transporter	3.81
*ythQ*	ABC transporter permease	3.46
*fadF*	iron-sulfur-binding reductase	3.13
*acdA*	acyl-CoA dehydrogenase	3.03
*fadH*	short chain dehydrogenase	2.96
*gltA*	glutamate synthase large subunit	2.95
*ythP*	ABC transporter ATP-binding protein	2.80
*fbpB*	hypothetical protein	2.67
*lcfB*	long-chain-fatty-acid--CoA ligase	2.62
*gltB*	glutamate synthase subunit beta	2.60
*ydjP*	peroxydase	2.60
*yfhM*	hydrolase	2.53
*dhbC*	isochorismate synthase DhbC	2.49
*ykuN*	flavodoxin	2.42
*ycnJ*	copper import protein	2.39
*etfB*	electron transfer flavoprotein subunit beta	2.37

**Fig 1:**
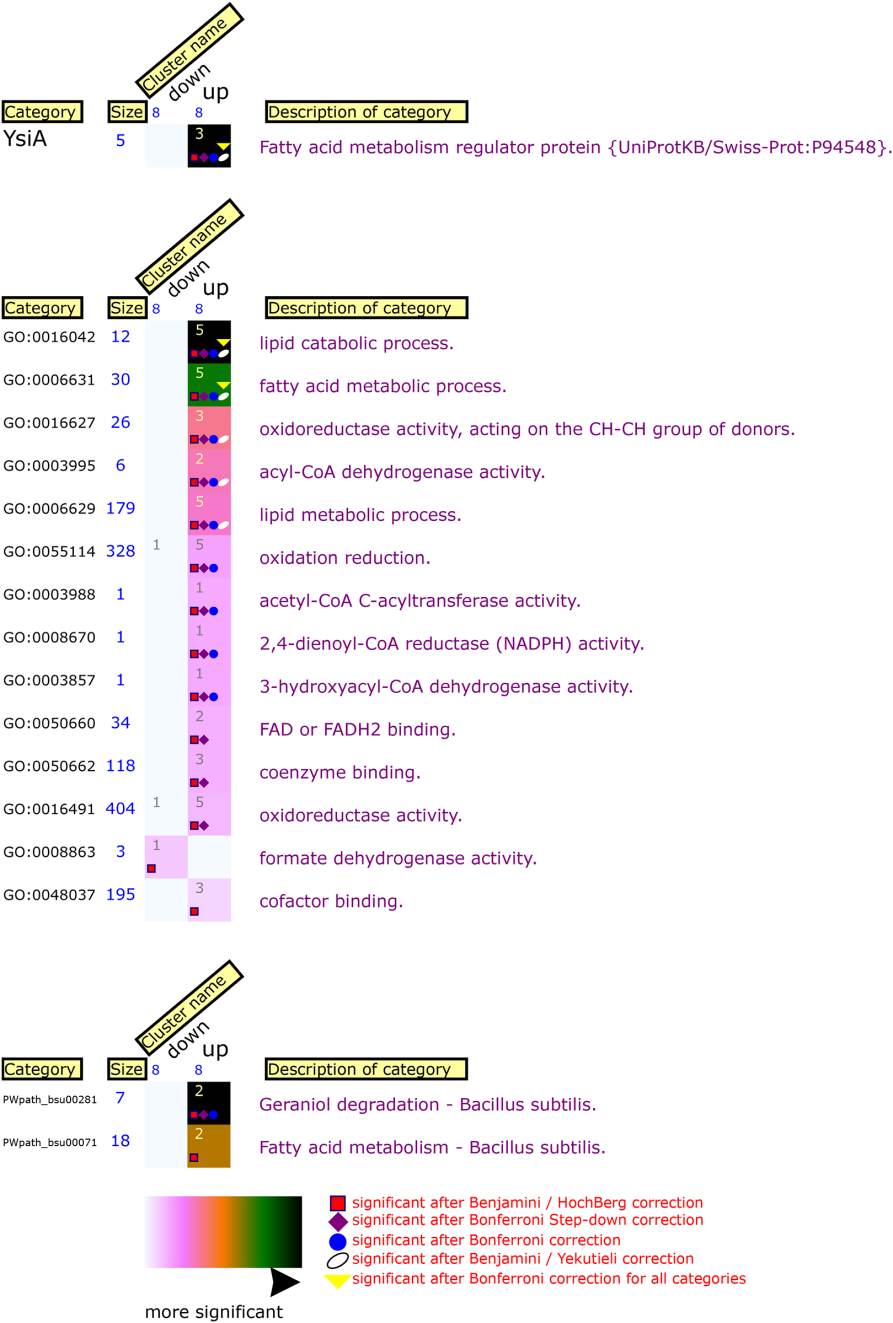
FIVA analysis of *B*. *subtilis* 168 *sigF*::*spc* gene expression under vegetative conditions. Genes from one DNA microarray dataset (*sigF* mutant strain compared to wild-type strain during exponential growth) were partitioned into up- and down-regulated clusters. The size of each cluster is displayed in blue underneath the cluster name. Numbers in each rectangle represent absolute values of occurrences. The significance of occurrences is visualized in a colour gradient that is displayed at the bottom of the plot. The description of each category is placed at the right. Multiple testing correction results are visualized using five different symbols to distinguish between the individual corrections. The number of symbols placed in each rectangle corresponds to the number of multiple testing corrections after which the annotation is found significant. This figure legend is cited from [33].

Under vegetative conditions the most extreme fold changes were -4.33 and 11.8, in contrast to the much higher expression ratios under sporulation-conditions that ranged from -385 to 10. The rest of the expression ratios under vegetative conditions was just under and above the set threshold of -2 and 2, respectively. Although the transcript ratios determined in these microarray experiments do not give information about absolute transcript numbers, in comparison with the observed fold changes under sporulation conditions the expression ratios under vegetative conditions are of a substantially lower magnitude. In addition to the fact that the number of differentially expressed genes is almost ten-fold lower than under sporulation conditions, this suggests that the *sigF* mutation does not have a profound effect under vegetative conditions. This is not unexpected since SigF is known as a sporulation-specific sigma factor. Among the down-regulated genes, the highest expression change occurred for *yosX* (-4.33; Table 3). The expression change of other genes ranged from -3.61 to -2 fold change. The *sigF* operon and some sporulation genes regulated by SigF, such as *spoIIQ*, were down-regulated which indicates that sporulation probably was initiated to a small extent in the wild-type strain. The majority of the down-regulated genes were associated with anabolism, for example, the *ilv* and *leu* genes involved in branched-chain amino acid synthesis [41] and the *pur* genes involved in synthesis of purines [42]. These genes are repressed in the presence of the products they synthesize [42,43]. The difference in relative transcript levels between the *sigF* mutant strain and the wild-type strain, might suggest that higher levels of these compounds are present in the *sigF* mutant culture. Similar to the down-regulated genes, the expression change of most up-regulated genes lies just above the cut-off of 2-fold. Many genes with a fold change higher than that are involved with fatty acid metabolism (*acdA*, *etfB*, *fadAEFHN*, *lcfB*). The highest up-regulated gene is *yxeD* (11.8 fold) whose function is unknown [20,40]. BLAST searches (http://blast.ncbi.nlm.nih.gov/Blast.cgi) resulted in purely hypothetical proteins. In general the up-regulated genes are involved in catabolism. Functional analysis with FIVA shows that fatty acid metabolism—and in particular, degradation—is overrepresented among the genes with changed expression (Fig 1).

Some of the fatty acid degradation genes that were up-regulated in the *sigF* mutant strain under vegetative conditions were up-regulated under sporulation conditions, too. This suggests that their regulation might be linked to sporulation- or *sigF*. It is known that upon sporulation the operon *fadAEN*, belonging to the FadR regulon, can be induced by Sdp (sporulation delay protein) [44]. However, the *sdpABC* operon was down-regulated in the *sigF* mutant strain and thus not likely to be involved in the induction of the fatty acid degradation genes in this case. In our case, not only *fadAEN*, but the complete FadR regulon was up-regulated under vegetative conditions. This regulon is subject to carbon catabolite repression [45] and induced by long-chain fatty acids [46]. Therefore it is more likely that one of these factors play a role in the observed up-regulation of fatty acid degradation genes under vegetative conditions. On the other hand, no other genes under carbon catabolite repression were induced under exponential conditions, making a role for CCR relief in the induction of the FadR regulon unlikely. The knowledge that the *sigF* mutant and wild-type strain were grown in glucose-free TY medium and were harvested at the same OD and time-point, diminishes the potential role of CCR relief in FadR regulon induction even more. Repression by FadR is antagonized by long-chain acyl Coa’s, in particular derivatives of 12-metyltetradecanoic and 13-metyltetradecanoic acids (anteisoC15:0 and isoC15:0 branched-chain fatty acids) [46], which are the most abundant fatty acids in the cell [47]. As suggested previously by Koburger et al. [48], cell lysis and membrane turnover might provide branched chained fatty acids synthesized in growing cells, thereby inducing the FadR regulon. The phenotype of the *sigF* mutant strain potentially leads to increased lysis in comparison to the wild-type strain. Cells that have initiated sporulation, but lack a functional *sigF* gene are unable to complete forespore differentiation at stage II of the sporulation process and instead form an additional polar septum at the opposite pole [17]. As described by Dworkin et al. [18], these disporic sporangias are able to reinitiate growth from both polar compartments when excess of nutrients is available. During this process the mother cell lyses and possibly releases cell components including long chain fatty acids into the environment. Thus, if some cells initiate sporulation under vegetative conditions the arrest at stage II of the sporulation process could be the cause of the observed transcriptome differences. Occasional observation of disporic sporangia under the microscope (≤0.1%) in samples from vegetative growing cultures supports this hypothesis.

Considering this, the observed transcriptome changes in this study are more likely an effect of cellular and physiological conditions resulting from arrest at sporulation stage II rather than a direct effect of gene regulation by SigF. The exact cause of the transcriptome change in the *sigF* mutant strain needs to be deciphered by further experimental work, including determining whether there is lysis or outgrowth of disporic sporangia occurs, and whether fatty acid concentrations become high enough to induce the FadR regulon.

### Concluding remarks

In this study a transcriptomic comparison of a *B*. *subtilis sigF* deletion strain to a wild-type strain is performed during vegetative growth. In agreement with the fact that σF is a sporulation-specific sigma factor, a relative small amount of genes are found to be differentially expressed under non-sporulation conditions. Although some up-regulation of genes involved in catabolism—most notably fatty acid degradation genes—and some down-regulation of genes involved in anabolism was observed, this study shows that the *sigF* mutation has a minor effect on the transcriptome under vegetative conditions. The changes possibly reflect metabolic effects caused by minor lysis of cells that have initiated sporulation, and occasional outgrowth of disporic sporangia.

## Supporting Information

S1 Fig FIVA analysis of *B*. *subtilis* 168 *sigF*::*spc* gene expression under sporulation conditions. (TIF)

S1 Table Top 200 genes down-regulated in *B*. *subtilis* 168 *sigF*::*spc* under sporulation conditions. (DOCX)

S2 Table Top 200 genes up-regulated in *B*. *subtilis* 168 *sigF*::*spc* under sporulation conditions. (DOCX)

S3 Table Total genes down-regulated in *B*. *subtilis* 168 *sigF*::*spc* under non-sporulation conditions. (DOCX)

S4 Table Total genes up-regulated in *B*. *subtilis* 168 *sigF*::*spc* under non-sporulation conditions. (DOCX)
