# Supplementary figures and images for: Transcriptional Profile of Bacillus subtilis sigF-Mutant during Vegetative Growth
Source: PLoS One. 2015 Oct 27;10(10):e0141553. doi: 10.1371/journal.pone.0141553 (PMC4624776; doi:10.1371/journal.pone.0141553)

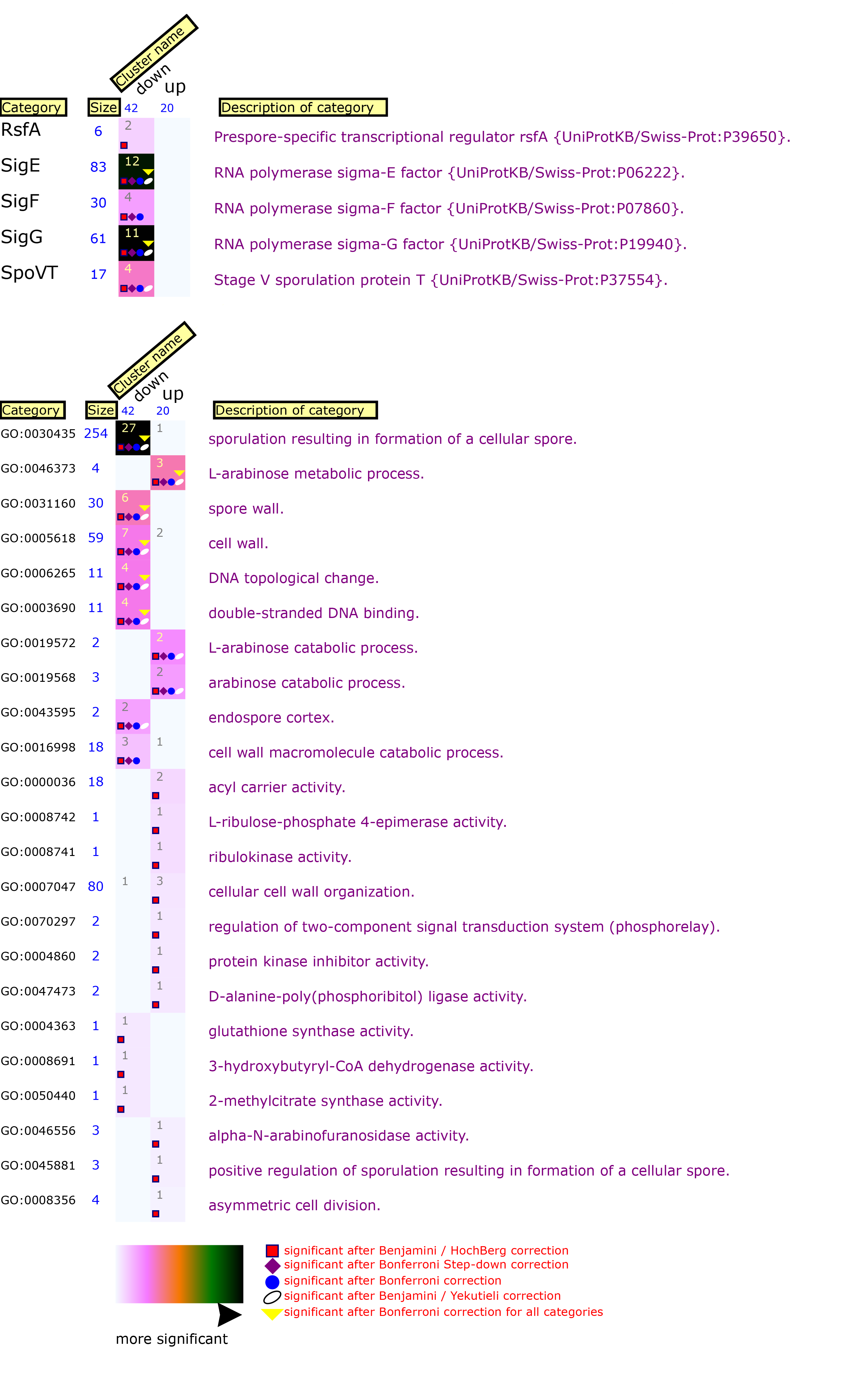

Supplement: S1 Fig — (TIF) [file pone.0141553.s001.tif]
