# Supplementary material for: Transcriptional Profile of Bacillus subtilis sigF-Mutant during Vegetative Growth
Source: PLoS One. 2015 Oct 27;10(10):e0141553. doi: 10.1371/journal.pone.0141553 (PMC4624776; doi:10.1371/journal.pone.0141553)
Supplement: S1 Table — (DOCX) [file pone.0141553.s002.docx]

| **Name** | **product** | **Fold** | **Bayes.p** |
| --- | --- | --- | --- |
| *spoIIQ* | stage II sporulation protein Q | -385.01 | 2.00E-15 |
| *spoIIID* | stage III sporulation protein D | -340.68 | 6.24E-12 |
| *ythC* | not found | -239.55 | 1.49E-14 |
| *safA* | spoivd-associated factor A | -225.29 | 0 |
| *sspE* | small acid-soluble spore protein gamma-type | -209.73 | 0 |
| *sspA* | small acid-soluble spore protein A | -202.34 | 7.77E-15 |
| *sspB* | small acid-soluble spore protein B | -200.00 | 2.91E-14 |
| *gdh* | glucose 1-dehydrogenase | -164.09 | 3.10E-08 |
| *spoVID* | stage VI sporulation protein D | -163.22 | 1.78E-15 |
| *cotE* | spore coat protein E | -159.91 | 7.77E-15 |
| *cwlJ* | cell wall hydrolase CwlJ | -149.63 | 1.25E-14 |
| *usd* | protein usd | -147.87 | 2.85E-11 |
| *yheC* | endospore coat-associated protein YheC | -136.27 | 1.69E-13 |
| *cydC* | ATP-binding/permease protein CydC | -127.84 | 1.21E-07 |
| *yhcN* | lipoprotein | -122.14 | 5.68E-10 |
| *coxA* | sporulation cortex protein CoxA | -111.33 | 9.09E-12 |
| *spoVR* | stage V sporulation protein R | -98.54 | 7.41E-13 |
| *mmgB* | 3-hydroxybutyryl-CoA dehydrogenase | -96.15 | 3.66E-11 |
| *spoVK* | stage V sporulation protein K | -87.20 | 2.68E-06 |
| *prkA* | serine protein kinase PrkA | -84.91 | 8.60E-12 |
| *sspC* | small acid-soluble spore protein C | -83.23 | 0 |
| *glnH* | ABC transporter glutamine-binding protein GlnH | -82.45 | 2.49E-10 |
| *yqfX* | hypothetical protein | -81.76 | 1.46E-12 |
| *ydhD* | sporulation protein | -74.89 | 9.84E-10 |
| *yhcV* | CBS domain-containing protein YhcV | -74.87 | 3.60E-12 |
| *yxeD* | hypothetical protein | -73.18 | 1.90E-09 |
| *cotJC* | spore coat peptide assembly protein CotJC | -72.82 | 2.22E-16 |
| *spoIVA* | stage IV sporulation protein A | -67.44 | 1.61E-14 |
| *sspD* | small acid-soluble spore protein D | -66.64 | 1.06E-11 |
| *yhcQ* | spore coat protein F | -66.63 | 1.24E-12 |
| *spoIIIC* | RNA polymerase sporulation-specific sigma factor K | -64.08 | 9.25E-09 |
| *cotJB* | spore coat protein CotJB | -61.74 | 4.44E-16 |
| *cydB* | cytochrome d ubiquinol oxidase subunit 2 | -61.08 | 4.96E-09 |
| *yodP* | N-acetyltransferase YodP | -59.87 | 9.66E-11 |
| *ytzC* | hypothetical protein | -58.68 | 7.04E-12 |
| *cotT* | spore coat protein | -54.63 | 2.01E-10 |
| *yjfA* | hypothetical protein | -53.40 | 6.23E-13 |
| *ymfJ* | hypothetical protein | -52.20 | 3.22E-13 |
| *mmgD* | 2-methylcitrate synthase | -49.79 | 1.13E-12 |
| *sspJ* | small acid-soluble spore protein J | -49.16 | 8.61E-10 |
| *rsfA* | prespore-specific transcriptional regulator RsfA | -46.20 | 6.36E-11 |
| *yheD* | endospore coat-associated protein YheD | -45.84 | 2.21E-12 |
| *yfhD* | hypothetical protein | -45.57 | 1.00E-12 |
| *yutG* | hypothetical protein | -45.47 | 1.76E-10 |
| *cotF* | spore coat protein F | -44.37 | 7.55E-10 |
| *yngF* | enoyl-CoA hydratase/isomerase YngF | -43.67 | 1.99E-09 |
| *yodR* | coenzyme A transferase subunit beta | -41.97 | 6.14E-10 |
| *ykuS* | hypothetical protein | -39.09 | 4.80E-14 |
| *gerBC* | spore germination protein B3 | -38.79 | 5.75E-06 |
| *mmgC* | acyl-CoA dehydrogenase | -38.62 | 5.96E-12 |
| *ytfJ* | spore protein YtfJ | -38.48 | 1.13E-09 |
| *yugT* | oligo-1,6-glucosidase 3 | -37.86 | 9.84E-09 |
| *ysnD* | hypothetical protein | -36.79 | 1.34E-07 |
| *yozN* | hypothetical protein | -36.46 | 1.29E-14 |
| *yxjF* | oxidoreductase | -36.40 | 1.50E-10 |
| *yhfM* | hypothetical protein | -36.17 | 3.90E-10 |
| *yqfT* | hypothetical protein | -35.77 | 3.78E-10 |
| *yflJ* | hypothetical protein | -35.48 | 9.60E-13 |
| *ylbO* | hypothetical protein | -34.55 | 5.62E-10 |
| *yngG* | hydroxymethylglutaryl-CoA lyase YngG | -33.53 | 1.84E-10 |
| *yhfW* | rieske 2Fe-2S iron-sulfur protein YhfW | -33.07 | 4.03E-11 |
| *yesJ* | N-acetyltransferase YesJ | -32.92 | 2.29E-14 |
| *glnM* | glutamine ABC transporter permease protein GlnM | -32.88 | 9.36E-08 |
| *yxjC* | transporter | -32.69 | 5.49E-10 |
| *yndA* | hypothetical protein | -31.52 | 5.61E-11 |
| *yqfC* | hypothetical protein | -31.40 | 1.95E-10 |
| *yaaH* | spore germination protein YaaH | -31.08 | 2.38E-10 |
| *sspN* | small acid-soluble spore protein N | -30.54 | 5.11E-08 |
| *spoIVCA* | DNA recombinase | -30.48 | 1.94E-10 |
| *yqzG* | hypothetical protein | -30.48 | 1.45E-10 |
| *yocN* | hypothetical protein | -30.46 | 2.07E-13 |
| *yveA* | aspartate-proton symporter | -30.04 | 1.88E-08 |
| *miaA* | tRNA dimethylallyltransferase | -29.66 | 7.93E-11 |
| *yhdB* | hypothetical protein | -29.02 | 1.32E-08 |
| *yraE* | hypothetical protein | -29.01 | 2.02E-08 |
| *tlp* | small acid-soluble spore protein Tlp | -28.86 | 4.34E-08 |
| *ysxE* | hypothetical protein | -28.71 | 3.66E-11 |
| *yraG* | spore coat protein F | -28.67 | 1.32E-07 |
| *yjdH* | hypothetical protein | -28.62 | 1.26E-12 |
| *glnQ* | glutamine transport ATP-binding protein GlnQ | -27.74 | 1.24E-09 |
| *lonB* | Lon protease 2 | -27.55 | 2.13E-10 |
| *spoIIP* | stage II sporulation protein P | -27.50 | 6.18E-10 |
| *yhxC* | oxidoreductase | -27.17 | 3.70E-09 |
| *yumB* | NADH dehydrogenase-like protein YumB | -27.12 | 2.09E-09 |
| *cypA* | cytochrome P450 | -26.27 | 9.29E-08 |
| *yraD* | spore coat protein F | -26.24 | 1.56E-07 |
| *yrrD* | hypothetical protein | -25.78 | 4.60E-11 |
| *ythA* | cytochrome bd menaquinol oxidase subunit I | -25.19 | 1.15E-07 |
| *yybI* | hypothetical protein | -24.51 | 2.62E-11 |
| *spoIIR* | stage II sporulation protein R | -24.45 | 9.37E-11 |
| *yitF* | isomerase | -24.22 | 2.60E-06 |
| *yraF* | spore coat protein F | -24.03 | 3.09E-08 |
| *yhbH* | stress response UPF0229 protein YhbH | -24.02 | 1.91E-08 |
| *yfjS* | polysaccharide deacetylase PdaA | -23.91 | 1.41E-08 |
| *yjcA* | sporulation protein YjcA | -23.42 | 1.59E-09 |
| *yobW* | mother cell-specific membrane sporulation protein | -23.26 | 7.41E-09 |
| *mpr* | extracellular metalloprotease | -23.09 | 2.45E-13 |
| *yqxA* | hypothetical protein | -23.02 | 1.70E-07 |
| *spoIVB* | peptidase S55 | -22.77 | 9.14E-11 |
| *nadA* | quinolinate synthase A | -22.65 | 6.22E-11 |
| *yngE* | carboxylase YngE | -22.60 | 8.99E-10 |
| *ywdL* | spore coat protein GerQ | -22.58 | 1.89E-09 |
| *ymfE* |  | -22.46 | 1.18E-08 |
| *phoB* | alkaline phosphatase 3 | -22.38 | 1.52E-09 |
| *yeeK* | spore coat protein YeeK | -22.35 | 2.64E-09 |
| *adhB* | zinc-type alcohol dehydrogenase | -22.10 | 2.70E-06 |
| *yodS* | coenzyme A transferase subunit alpha | -21.89 | 1.42E-11 |
| *ytlD* | ABC transporter permease | -21.58 | 7.18E-10 |
| *yteA* | hypothetical protein | -21.53 | 2.73E-07 |
| *yckD* | hypothetical protein | -21.49 | 1.09E-08 |
| *sspP* | small acid-soluble spore protein P | -21.36 | 3.90E-07 |
| *yurZ* | hypothetical protein | -21.28 | 3.10E-09 |
| *ydcC* | sporulation protein YdcC | -21.25 | 1.50E-13 |
| *ysnE* | N-acetyltransferase YsnE | -21.16 | 5.14E-08 |
| *yjcB* |  | -21.11 | 2.11E-08 |
| *ynzB* | hypothetical protein | -21.04 | 5.19E-09 |
| *yhaX* | hydrolase | -20.80 | 2.79E-12 |
| *yxeE* | hypothetical protein | -20.74 | 7.54E-06 |
| *yqiQ* | methylisocitrate lyase | -20.64 | 1.16E-13 |
| *yjbX* | spore coat protein O | -20.41 | 1.62E-10 |
| *gerM* | spore germination protein GerM | -20.15 | 6.32E-12 |
| *yknU* | ABC transporter ATP-binding protein | -19.88 | 9.93E-11 |
| *yozE* | hypothetical protein | -19.62 | 6.22E-10 |
| *sspK* | small acid-soluble spore protein K | -19.52 | 2.08E-07 |
| *yngI* | acyl-CoA synthetase YngI | -19.24 | 3.92E-09 |
| *yknV* | ABC transporter ATP-binding protein | -18.70 | 4.06E-11 |
| *spmA* | spore maturation protein A | -18.47 | 2.83E-06 |
| *gerKA* | spore germination protein KA | -18.36 | 2.94E-06 |
| *yqzE* | hypothetical protein | -18.28 | 2.02E-07 |
| *yunA* | L-Ala--D-Glu endopeptidase | -18.22 | 1.61E-08 |
| *ypeB* | sporulation protein YpeB | -18.13 | 4.71E-08 |
| *cotH* | inner spore coat protein H | -18.04 | 9.23E-07 |
| *katX* | catalase X | -18.00 | 7.28E-12 |
| *spsK* | spore coat polysaccharide biosynthesis protein SpsK | -17.89 | 1.40E-09 |
| *ykoS* | membrane protein | -17.88 | 0.000109521 |
| *spoIIIAH* | stage III sporulation protein AH | -17.66 | 2.24E-11 |
| *cotJA* | spore coat associated protein CotJA | -17.63 | 4.98E-13 |
| *ywbG* | hypothetical protein | -17.51 | 1.93E-05 |
| *yjbA* | hypothetical protein | -17.28 | 1.69E-12 |
| *yfhE* | hypothetical protein | -17.14 | 9.44E-10 |
| *gerBB* | spore germination protein B2 | -16.82 | 1.13E-06 |
| *sspO* | small acid-soluble spore protein O | -16.57 | 2.49E-06 |
| *yuzA* | membrane protein | -16.51 | 5.66E-09 |
| *spoIIIAG* | stage III sporulation protein AG | -16.50 | 1.27E-10 |
| *ydhU* |  | -16.11 | 2.41E-09 |
| *ytaA* | spore coat protein I | -15.89 | 1.48E-05 |
| *yhaL* | sporulation protein YhaL | -15.42 | 8.85E-09 |
| *citH* | citrate transporter | -15.24 | 1.89E-09 |
| *ymxH* | hypothetical protein | -15.08 | 2.23E-12 |
| *cydD* | ATP-binding/permease protein CydD | -15.02 | 4.99E-06 |
| *ytxC* | hypothetical protein | -14.98 | 3.57E-13 |
| *yrbG* | hypothetical protein | -14.55 | 1.12E-09 |
| *ywbA* | permease IIC component YwbA | -14.47 | 7.31E-07 |
| *spoIIIAF* | stage III sporulation protein AF | -14.39 | 8.06E-10 |
| *yuiC* | hypothetical protein | -14.36 | 1.35E-11 |
| *ykzE* | hypothetical protein | -14.35 | 3.83E-08 |
| *bioW* | 6-carboxyhexanoate--CoA ligase | -14.34 | 2.74E-10 |
| *bioA* | L-lysine-8-amino-7-oxononanoate aminotransferase | -13.90 | 1.89E-12 |
| *ywlB* | hypothetical protein | -13.72 | 7.22E-09 |
| *yngH* | biotin carboxylase 2 | -13.68 | 1.87E-10 |
| *yozP* | hypothetical protein | -13.53 | 9.41E-09 |
| *yitG* | MFS transporter | -13.38 | 5.15E-08 |
| *yrzK* | hypothetical protein | -13.22 | 6.42E-08 |
| *yhdC* | hypothetical protein | -13.21 | 3.73E-09 |
| *rapC* | response regulator aspartate phosphatase C | -13.12 | 2.05E-13 |
| *mmgE* | 2-methylcitrate dehydratase | -13.10 | 2.12E-10 |
| *bcsA* | chalcone synthase | -13.07 | 4.25E-07 |
| *yufS* | hypothetical protein | -13.05 | 5.85E-10 |
| *yrbD* | sodium/proton-dependent alanine carrier protein YrbD | -13.04 | 2.56E-09 |
| *yknT* | sporulation protein cse15 | -13.01 | 2.69E-10 |
| *cotM* | spore coat protein M | -12.96 | 0.000322905 |
| *sspF* | small acid-soluble spore protein SspF | -12.91 | 2.40E-07 |
| *kamA* | L-lysine 2,3-aminomutase | -12.84 | 6.82E-13 |
| *yqhV* | hypothetical protein | -12.71 | 4.01E-11 |
| *yusN* | hypothetical protein | -12.65 | 1.99E-09 |
| *sodF* | superoxide dismutase | -12.62 | 2.66E-12 |
| *ytzH* | hypothetical protein | -12.44 | 5.51E-09 |
| *spsE* | spore coat polysaccharide biosynthesis protein SpsE | -12.32 | 1.65E-07 |
| *glcU* | glucose uptake protein GlcU | -12.25 | 6.48E-09 |
| *yusW* | hypothetical protein | -12.23 | 2.90E-08 |
| *yqhG* | hypothetical protein | -12.08 | 1.10E-08 |
| *spoVFB* | dipicolinate synthase subunit B | -12.02 | 5.56E-07 |
| *yngL* | hypothetical protein | -11.99 | 2.86E-06 |
| *yqcI* | hypothetical protein | -11.81 | 5.73E-07 |
| *ymaF* | hypothetical protein | -11.57 | 1.72E-08 |
| *ylbJ* | sporulation integral membrane protein YlbJ | -11.44 | 0.006842119 |
| *spsJ* | dTDP-glucose 4,6-dehydratase | -11.39 | 6.39E-07 |
| *yoaQ* | hypothetical protein | -11.37 | 2.32E-08 |
| *yjmC* | oxidoreductase | -11.35 | 5.47E-10 |
| *yckC* | hypothetical protein | -11.25 | 6.16E-08 |
| *yqfD* | hypothetical protein | -11.06 | 3.89E-09 |
| *yisY* | AB hydrolase superfamily protein YisY | -11.02 | 1.80E-08 |
| *ypfB* | hypothetical protein | -11.00 | 3.47E-09 |
| *ykpC* | hypothetical protein | -10.88 | 2.91E-10 |
| *ybfJ* | hypothetical protein | -10.85 | 8.43E-10 |
| *scoB* | succinyl-CoA:3-ketoacid coenzyme A transferase subunit B | -10.85 | 2.00E-07 |
| *spoIIIAA* | stage III sporulation protein AA | -10.84 | 3.51E-12 |
| *yngD* | oligoribonuclease NrnB | -10.83 | 2.47E-07 |
| *spoVT* | stage V sporulation protein T | -10.72 | 3.58E-09 |
