# Supplementary material for: Transcriptional Profile of Bacillus subtilis sigF-Mutant during Vegetative Growth
Source: PLoS One. 2015 Oct 27;10(10):e0141553. doi: 10.1371/journal.pone.0141553 (PMC4624776; doi:10.1371/journal.pone.0141553)
Supplement: S2 Table — (DOCX) [file pone.0141553.s003.docx]

| **Name** | **product** | **fold** | **Bayes.p** |
| --- | --- | --- | --- |
| *yqbS* | hypothetical protein | 9.63 | 2.61E-02 |
| *yvfU* | transcriptional regulator | 6.24 | 1.34E-02 |
| *ftsX* | cell division protein FtsX | 5.95 | 5.73E-04 |
| *araQ* | L-arabinose transport system permease protein AraQ | 5.18 | 3.17E-09 |
| *yonB* | hypothetical protein | 5.06 | 9.07E-05 |
| *yomY* | hypothetical protein | 4.70 | 5.99E-09 |
| *yonD* | hypothetical protein | 4.53 | 6.69E-08 |
| *sda* | sporulation inhibitor sda | 4.31 | 1.81E-05 |
| *yonT* | hypothetical protein | 4.27 | 6.04E-06 |
| *yonC* | hypothetical protein | 4.20 | 1.23E-07 |
| *abfA* | alpha-L-arabinofuranosidase | 4.19 | 2.24E-08 |
| *araL* | arabinose operon protein AraL | 4.01 | 1.40E-09 |
| *araP* | L-arabinose transport system permease protein | 4.00 | 5.23E-08 |
| *araD* | L-ribulose-5-phosphate 4-epimerase | 3.90 | 1.13E-07 |
| *cwlO* | peptidoglycan DL-endopeptidase CwlO | 3.89 | 1.75E-08 |
| *yoqM* | hypothetical protein | 3.73 | 7.08E-07 |
| *yonX* | hypothetical protein | 3.72 | 2.74E-04 |
| *dltC* | D-alanine--poly(phosphoribitol) ligase subunit 2 | 3.65 | 9.52E-07 |
| *dhbF* | dimodular nonribosomal peptide synthase | 3.52 | 1.37E-06 |
| *yomZ* | hypothetical protein | 3.50 | 1.44E-06 |
| *lytE* | peptidoglycan endopeptidase LytE | 3.46 | 1.99E-08 |
| *araB* | ribulokinase | 3.46 | 1.82E-09 |
| *yomT* | hypothetical protein | 3.40 | 2.19E-06 |
| *cheR* | chemotaxis protein methyltransferase | 3.38 | 3.25E-02 |
| *araA* | L-arabinose isomerase | 3.37 | 1.09E-08 |
| *yorH* | hypothetical protein | 3.36 | 8.17E-06 |
| *nrdF* | ribonucleoside-diphosphate reductase subunit beta | 3.35 | 4.43E-07 |
| *frlO* | ABC transporter extracellular-binding protein YurO | 3.27 | 1.27E-06 |
| *ydcT* | hypothetical protein | 3.26 | 6.12E-06 |
| *araN* | arabinose-binding protein | 3.22 | 2.82E-06 |
| *nrdE* | ribonucleoside-diphosphate reductase subunit alpha | 3.21 | 6.94E-09 |
| *gltT* | proton/sodium-glutamate symport protein | 3.19 | 3.27E-07 |
| *yqdB* | hypothetical protein | 3.17 | 2.49E-07 |
| *yonA* | hypothetical protein | 3.15 | 8.52E-06 |
| *ydcR* | DNA relaxase NicK | 3.14 | 2.90E-04 |
| *dltB* | protein DltB | 3.10 | 7.01E-07 |
| *yomW* | hypothetical protein | 3.07 | 3.01E-06 |
| *yhdH* | sodium-dependent transporter YhdH | 3.03 | 5.76E-04 |
| *dltD* | poly D-alanine transfer protein DltD | 3.01 | 4.98E-06 |
| *dhbE* | 2,3-dihydroxybenzoate-AMP ligase | 2.97 | 1.68E-06 |
| *ilvD* | dihydroxy-acid dehydratase | 2.97 | 3.34E-08 |
| *yqzH* | hypothetical protein | 2.96 | 1.26E-03 |
| *nhaX* | stress response protein NhaX | 2.94 | 1.59E-05 |
| *dhbB* | isochorismatase | 2.84 | 1.20E-05 |
| *ykuG* |  | 2.84 | 1.34E-05 |
| *yomX* | hypothetical protein | 2.80 | 2.18E-05 |
| *yosB* | hypothetical protein | 2.80 | 1.48E-02 |
| *yomS* | hypothetical protein | 2.79 | 8.35E-07 |
| *gsiB* | glucose starvation-inducible protein B | 2.76 | 1.17E-05 |
| *yhcR* | endonuclease | 2.72 | 1.19E-07 |
| *oppC* | oligopeptide transport system permease protein OppC | 2.72 | 2.89E-07 |
| *ydjN* | hypothetical protein | 2.70 | 1.87E-05 |
| *yonN* | DNA-binding protein HU 2 | 2.69 | 1.71E-06 |
| *ywtD* | gamma-dl-glutamyl hydrolase | 2.69 | 2.25E-06 |
| *yonH* | hypothetical protein | 2.68 | 1.03E-05 |
| *minD* | septum site-determining protein MinD | 2.66 | 2.79E-06 |
| *oppD* | oligopeptide transport ATP-binding protein OppD | 2.66 | 1.56E-07 |
| *abnA* | arabinan-endo 1,5-alpha-L-arabinase | 2.65 | 8.31E-08 |
| *yozM* | prophage-derived-like protein YozM | 2.65 | 1.22E-03 |
| *yurM* | ABC transporter permease | 2.64 | 6.50E-05 |
| *alaT* | aminotransferase YugH | 2.63 | 4.10E-05 |
| *yjcM* | hypothetical protein | 2.63 | 9.78E-06 |
| *pycA* | pyruvate carboxylase | 2.61 | 2.72E-07 |
| *oppF* | oligopeptide transport ATP-binding protein OppF | 2.60 | 7.93E-06 |
| *yosE* | membrane protein | 2.57 | 3.32E-04 |
| *yomO* | hypothetical protein | 2.55 | 1.86E-04 |
| *ydcS* | hypothetical protein | 2.55 | 4.33E-04 |
| *ykuF* | 2,4-dienoyl-CoA reductase | 2.53 | 3.70E-06 |
| *yfmR* | ABC transporter ATP-binding protein | 2.53 | 1.71E-06 |
| *yosQ* | HNH homing endonuclease YosQ | 2.52 | 1.54E-05 |
| *yoqA* | hypothetical protein | 2.50 | 1.42E-04 |
| *acpK* | polyketide biosynthesis acyl-carrier-protein AcpK | 2.50 | 7.72E-07 |
| *yorZ* | hypothetical protein | 2.50 | 1.65E-03 |
| *yfmS* | sensory transducer protein YfmS | 2.50 | 5.57E-05 |
| *nprE* | bacillolysin | 2.47 | 1.14E-06 |
| *ywbN* | deferrochelatase/peroxidase EfeN | 2.45 | 9.19E-06 |
| *csbD* | stress response protein CsbD | 2.44 | 2.05E-05 |
| *ydcO* | hypothetical protein | 2.44 | 7.21E-06 |
| *fliH* | flagellar assembly protein FliH | 2.44 | 1.50E-05 |
| *ezrA* | septation ring formation regulator EzrA | 2.44 | 3.20E-05 |
| *yomN* | hypothetical protein | 2.43 | 5.62E-04 |
| *fliF* | flagellar M-ring protein | 2.43 | 3.56E-06 |
| *yhcS* | hypothetical protein | 2.43 | 8.37E-06 |
| *yonG* | hypothetical protein | 2.43 | 3.83E-03 |
| *yorD* | stress response protein SCP1 | 2.42 | 1.79E-05 |
| *sucC* | succinyl-CoA ligase [ADP-forming] subunit beta | 2.41 | 1.47E-06 |
| *leuB* | 3-isopropylmalate dehydrogenase | 2.41 | 5.65E-06 |
| *yceC* | stress response protein SCP2 | 2.41 | 6.50E-07 |
| *spo0E* | aspartyl-phosphate phosphatase Spo0E | 2.40 | 9.03E-06 |
| *dnaN* | DNA polymerase III subunit beta | 2.40 | 3.07E-05 |
| *alsT* | amino acid carrier protein AlsT | 2.40 | 1.39E-04 |
| *yonK* | hypothetical protein | 2.40 | 3.17E-03 |
| *ftsE* | cell division ATP-binding protein FtsE | 2.39 | 2.83E-05 |
| *yvrO* | ABC transporter ATP-binding protein | 2.38 | 2.29E-05 |
| *xkdH* | phage-like element PBSX protein XkdH | 2.38 | 3.48E-06 |
| *minC* | septum site-determining protein MinC | 2.38 | 1.37E-06 |
| *ybxG* | transporter | 2.37 | 1.35E-05 |
| *rsbV* | anti-sigma-B factor antagonist | 2.37 | 7.90E-08 |
| *ydaS* | hypothetical protein | 2.35 | 3.44E-06 |
| *yceF* | membrane protein | 2.35 | 5.39E-06 |
| *yomR* | hypothetical protein | 2.35 | 3.72E-05 |
| *motB* | motility protein B | 2.35 | 5.28E-06 |
| *trpC* | indole-3-glycerol phosphate synthase | 2.34 | 7.76E-04 |
| *trkA* | oxidoreductase | 2.33 | 4.86E-03 |
| *ydaL* | hypothetical protein | 2.33 | 1.52E-02 |
| *fliY* | flagellar motor switch phosphatase FliY | 2.33 | 2.29E-05 |
| *recA* | recombinase RecA | 2.32 | 3.38E-05 |
| *yisK* | hypothetical protein | 2.32 | 1.13E-06 |
| *fliJ* | flagellar FliJ protein | 2.32 | 4.43E-04 |
| *ylxF* | FlaA locus 22.9 kDa protein | 2.32 | 1.26E-05 |
| *yfmI* | MFS transporter | 2.32 | 7.91E-03 |
| *yflT* | general stress protein 17M | 2.31 | 1.75E-03 |
| *yusJ* | acyl-CoA dehydrogenase | 2.30 | 1.90E-08 |
| *ywgA* | hypothetical protein | 2.29 | 5.09E-06 |
| *fliK* | flagellar hook-length control protein | 2.29 | 3.64E-05 |
| *yurJ* | ABC transporter ATP-binding protein | 2.29 | 8.59E-06 |
| *flgE* | flagellar basal-body rod protein FlgG | 2.29 | 4.84E-06 |
| *dctP* | C4-dicarboxylate transport protein | 2.29 | 2.36E-05 |
| *gbsA* | betaine aldehyde dehydrogenase | 2.28 | 2.80E-05 |
| *yvrN* | ABC transporter permease | 2.28 | 3.00E-04 |
| *yvaX* | sporulation-delaying protein SdpB | 2.27 | 6.36E-04 |
| *ypmS* | hypothetical protein | 2.25 | 6.71E-05 |
| *yolB* | hypothetical protein | 2.25 | 5.12E-05 |
| *pbpD* | penicillin-binding protein 4 | 2.24 | 4.49E-05 |
| *pspA* | phage shock protein A homolog | 2.24 | 7.49E-06 |
| *cysC* | adenylyl-sulfate kinase | 2.24 | 2.52E-05 |
| *araE* | arabinose-proton symporter | 2.24 | 1.14E-05 |
| *ylxH* | flagellum site-determining protein YlxH | 2.24 | 1.26E-05 |
| *xylA* | xylose isomerase | 2.23 | 9.60E-06 |
| *dhbC* | isochorismate synthase DhbC | 2.23 | 8.18E-05 |
| *yorI* | hypothetical protein | 2.22 | 3.75E-05 |
| *yceE* | hypothetical protein | 2.22 | 8.38E-05 |
| *ytcP* | ABC transporter permease | 2.22 | 3.38E-04 |
| *yhfQ* | ABC transporter substrate-binding protein | 2.21 | 3.27E-05 |
| *yeeC* | hypothetical protein | 2.20 | 1.41E-03 |
| *penP* | beta-lactamase | 2.20 | 3.04E-04 |
| *yoqB* | hypothetical protein | 2.20 | 7.58E-04 |
| *ydaP* | thiamine pyrophosphate-containing protein YdaP | 2.19 | 5.27E-06 |
| *mreD* | rod shape-determining protein MreD | 2.18 | 1.39E-04 |
| *yomV* | hypothetical protein | 2.18 | 1.73E-05 |
| *pksG* | polyketide biosynthesis 3-hydroxy-3-methylglutaryl-ACP synthase PksG | 2.18 | 5.01E-06 |
| *ywbL* | ferrous iron permease EfeU | 2.17 | 1.37E-05 |
| *yteS* | lipoprotein | 2.16 | 4.12E-05 |
| *yurN* | ABC transporter permease | 2.16 | 3.30E-05 |
| *yurP* | fructosamine deglycase FrlB | 2.15 | 3.12E-05 |
| *ypmT* | hypothetical protein | 2.15 | 2.43E-05 |
| *yceD* | general stress protein 16U | 2.14 | 1.88E-06 |
| *yqeD* | hypothetical protein | 2.14 | 2.10E-02 |
| *uxaC* | uronate isomerase | 2.14 | 1.41E-04 |
| *ytcQ* | peptide ABC transporter substrate-binding protein | 2.14 | 1.38E-04 |
| *yuxK* | hypothetical protein | 2.13 | 4.11E-05 |
| *kdgK* | 2-dehydro-3-deoxygluconokinase | 2.13 | 2.13E-04 |
| *yddC* | hypothetical protein | 2.13 | 3.97E-05 |
| *yfiY* | siderophore-binding lipoprotein YfiY | 2.13 | 7.93E-07 |
| *tlpB* | methyl-accepting chemotaxis protein TlpB | 2.12 | 5.37E-03 |
| *ybcT* |  | 2.12 | 2.92E-05 |
| *ysnF* | stress response protein YsnF | 2.12 | 5.26E-04 |
| *yddA* | hypothetical protein | 2.12 | 2.65E-04 |
| *yorE* | hypothetical protein | 2.12 | 8.48E-05 |
| *fliT* | flagellar protein FliT | 2.11 | 4.63E-05 |
| *feuA* | iron-uptake system-binding protein | 2.11 | 5.18E-04 |
| *yopY* | hypothetical protein | 2.11 | 4.55E-04 |
| *yfiH* | hypothetical protein | 2.11 | 6.11E-05 |
| *ycnI* | hypothetical protein | 2.11 | 3.16E-05 |
| *yorG* | hypothetical protein | 2.10 | 4.89E-05 |
| *dacA* | D-alanyl-D-alanine carboxypeptidase DacA | 2.09 | 2.65E-05 |
| *asnB* | asparagine synthetase 1 | 2.09 | 3.16E-05 |
| *xsa* | alpha-L-arabinofuranosidase | 2.09 | 2.43E-05 |
| *appC* | oligopeptide transport system permease protein AppC | 2.09 | 3.22E-05 |
| *motA* | motility protein A | 2.09 | 8.74E-06 |
| *cheV* | chemotaxis protein CheV | 2.09 | 1.15E-05 |
| *yncM* | hypothetical protein | 2.09 | 4.12E-05 |
| *fliM* | flagellar motor switch protein FliM | 2.08 | 2.32E-04 |
| *ureB* | urease subunit beta | 2.08 | 1.63E-06 |
| *flgL* | flagellar hook-associated protein 3 | 2.08 | 5.25E-05 |
| *yfmT* | aldehyde dehydrogenase YfmT | 2.08 | 1.81E-05 |
| *yfnI* | lipoteichoic acid synthase | 2.08 | 4.02E-06 |
| *yhfF* | hypothetical protein | 2.08 | 5.99E-06 |
| *yomM* | recombinase-like protein YomM | 2.07 | 5.78E-03 |
| *yosP* | ribonucleoside-diphosphate reductase subunit beta | 2.07 | 2.03E-02 |
| *rsbW* | serine-protein kinase RsbW | 2.06 | 1.47E-06 |
| *fliD* | flagellar hook-associated protein 2 | 2.06 | 3.09E-05 |
| *yvfH* | L-lactate permease | 2.06 | 4.64E-06 |
| *flhF* | flagellar biosynthesis protein FlhF | 2.04 | 3.40E-04 |
| *ydgF* | transporter | 2.04 | 2.36E-03 |
| *yisL* | hypothetical protein | 2.04 | 1.90E-04 |
| *sucD* | succinyl-CoA ligase [ADP-forming] subunit alpha | 2.03 | 6.17E-06 |
| *mreBH* | rod-share determining protein MreBH | 2.03 | 5.31E-05 |
| *yvrP* | efflux system protein YvrP | 2.03 | 2.77E-05 |
| *acuC* | acetoin utilization protein AcuC | 2.02 | 6.03E-04 |
| *ureC* | urease subunit alpha | 2.02 | 2.30E-05 |
| *yoqS* | hypothetical protein | 2.02 | 2.96E-02 |
| *yonS* | lipoprotein | 2.01 | 1.52E-04 |
| *rsbX* | phosphoserine phosphatase RsbX | 2.01 | 1.62E-05 |
| *ydjK* | major myo-inositol transporter IolT | 2.01 | 8.71E-04 |
| *ycbB* | transcriptional regulatory protein GlnL | 2.01 | 6.36E-03 |
| *yqcF* | hypothetical protein | 2.01 | 4.66E-05 |
| *rapK* | response regulator aspartate phosphatase K | 2.00 | 1.31E-04 |
| *yopZ* | hypothetical protein | 2.00 | 1.44E-04 |
| *gtaB* | UTP--glucose-1-phosphate uridylyltransferase | 2.00 | 2.45E-06 |
