# Supplementary material for: Transcriptional Profile of Bacillus subtilis sigF-Mutant during Vegetative Growth
Source: PLoS One. 2015 Oct 27;10(10):e0141553. doi: 10.1371/journal.pone.0141553 (PMC4624776; doi:10.1371/journal.pone.0141553)
Supplement: S3 Table — (DOCX) [file pone.0141553.s004.docx]

| **Gene** | **Product** | **Fold change** | **Bayes. p-value** |
| --- | --- | --- | --- |
| *sigF* ^a^ | RNA polymerase sporulation-specific sigma factor F | -10.22 | 7.22E-09 |
| *yosX* | hypothetical protein | -4.33 | 4.17E-05 |
| *yjgC* | oxidoreductase | -3.61 | 1.52E-07 |
| *yjgD* | hypothetical protein | -3.38 | 2.75E-06 |
| *yotB* | metallo-dependent hydrolase | -3.36 | 3.36E-06 |
| *ysnF* | stress response protein | -3.17 | 5.50E-06 |
| *opuCA* | glycine betaine/carnitine/choline/choline sulfate ABC transporter ATP-binding protein | -2.91 | 9.87E-08 |
| *yknU* ^a^ | ABC transporter ATP-binding protein | -2.88 | 2.21E-07 |
| *spoIIAB* ^a^ | anti-sigma F factor | -2.87 | 1.39E-07 |
| *ygxB* | hypothetical protein | -2.83 | 7.38E-05 |
| *pbpX* | penicillin-binding endopeptidase X | -2.76 | 3.92E-05 |
| *yorO* | hypothetical protein | -2.70 | 1.63E-05 |
| *purH* | bifunctional phosphoribosylaminoimidazolecarboxamide formyltransferase/IMP cyclohydrolase | -2.70 | 6.83E-07 |
| *yotC* | hypothetical protein | -2.67 | 7.83E-05 |
| *yukC* | bacteriocin production protein | -2.67 | 8.11E-07 |
| *yflT* | heat stress induced protein | -2.58 | 5.40E-06 |
| *yhcW* ^a^ | phosphoglycolate phosphatase | -2.49 | 5.64E-04 |
| *comGF* | DNA transport platform protein | -2.49 | 6.50E-04 |
| *leuC* | isopropylmalate isomerase large subunit | -2.44 | 6.13E-07 |
| *purN* | phosphoribosylglycinamide formyltransferase | -2.44 | 1.09E-05 |
| *yotD* | hypothetical protein | -2.42 | 3.37E-04 |
| *yotH* | hypothetical protein | -2.41 | 1.61E-04 |
| *epsK* | membrane protein EpsK | -2.37 | 6.36E-03 |
| *sdpB* | sporulation-delaying protein SdpB | -2.35 | 2.37E-03 |
| *tdk* | thymidine kinase | -2.32 | 7.47E-04 |
| *rpmI* | 50S ribosomal protein L35 | -2.29 | 4.50E-05 |
| *leuB* | 3-isopropylmalate dehydrogenase | -2.29 | 2.50E-06 |
| *yobI* | membrane protein | -2.28 | 2.83E-02 |
| *yorN* | hypothetical protein | -2.28 | 3.62E-04 |
| *ykuL* | CBS domain-containing protein YkuL | -2.27 | 1.99E-03 |
| *pckA* | phosphoenolpyruvate carboxykinase | -2.26 | 6.85E-06 |
| *spoIIAA* ^a^ | anti-anti-sigma factor | -2.25 | 3.69E-06 |
| *leuD* | isopropylmalate isomerase small subunit | -2.25 | 1.43E-05 |
| *purD* | phosphoribosylamine--glycine ligase | -2.25 | 1.30E-06 |
| *pyrAA* | carbamoyl phosphate synthase small subunit | -2.24 | 5.07E-04 |
| *leuA* | 2-isopropylmalate synthase | -2.23 | 1.53E-05 |
| *yorQ* ^a^ | hypothetical protein | -2.22 | 3.34E-05 |
| *yybJ* | ATP-binding cassette protein | -2.22 | 4.85E-04 |
| *sdpA* | export of killing factor | -2.21 | 6.01E-04 |
| *yotG* | hypothetical protein | -2.19 | 1.07E-04 |
| *ywqK* | hypothetical protein | -2.18 | 2.92E-03 |
| *ywjC* | hypothetical protein | -2.17 | 1.59E-03 |
| *fbaA* | fructose-bisphosphate aldolase | -2.16 | 5.86E-06 |
| *comGC* | ComG operon protein 3 | -2.14 | 1.10E-02 |
| *acoB* | acetoin dehydrogenase E1 component subunit beta | -2.14 | 3.35E-05 |
| *acoA* | acetoin dehydrogenase E1 component subunit alpha | -2.13 | 8.81E-05 |
| *rnpA* | ribonuclease P | -2.12 | 7.03E-04 |
| *yotF* | hypothetical protein | -2.09 | 9.98E-04 |
| *yxiS* | hypothetical protein | -2.07 | 9.97E-05 |
| *epsE* | Glycosyltransferase family A | -2.07 | 6.16E-03 |
| *pyrH* | uridylate kinase | -2.06 | 3.01E-05 |
| *smc* | chromosome condensation and segregation SMC ATPase | -2.06 | 3.40E-04 |
| *ftsE* | cell division ATP-binding protein FtsE | -2.05 | 1.22E-03 |
| *ydaS* | hypothetical protein | -2.05 | 2.77E-03 |
| *comGA* | ComG operon protein 1 | -2.05 | 6.10E-03 |
| *yorM* | hypothetical protein | -2.05 | 3.54E-03 |
| *purM* | phosphoribosylaminoimidazole synthetase | -2.04 | 7.26E-05 |
| *spoIIQ* | stage II sporulation protein Q | -2.03 | 1.61E-03 |
| *ywqH* | hypothetical protein | -2.03 | 8.72E-03 |
| *ilvC* | ketol-acid reductoisomerase | -2.03 | 3.10E-04 |
| *tlpC* | methyl-accepting chemotaxis protein TlpC | -2.03 | 5.02E-03 |
| *ykuJ* ^a^ | hypothetical protein | -2.02 | 6.48E-04 |
| *bdbB* | disulfide bond formation protein B | -2.02 | 3.55E-03 |
| *katE* | catalase 2 | -2.01 | 7.58E-05 |
| *ohrB* | organic hydroperoxide resistance reductase B | -2.01 | 1.34E-04 |
| *ydaD* | short chain dehydrogenase | -2.01 | 1.21E-04 |
| *yorP* | hypothetical protein | -2.00 | 3.82E-05 |

^a^ also down-regulated under sporulation conditions
