# Supplementary material for: Transcriptional Profile of Bacillus subtilis sigF-Mutant during Vegetative Growth
Source: PLoS One. 2015 Oct 27;10(10):e0141553. doi: 10.1371/journal.pone.0141553 (PMC4624776; doi:10.1371/journal.pone.0141553)
Supplement: S4 Table — (DOCX) [file pone.0141553.s005.docx]

| **Gene** | **Product** | **Fold change** | **Bayes. p-value** |
| --- | --- | --- | --- |
| *yxeD* | hypothetical protein | 11.84 | 3.85E-02 |
| *fadE* ^a^ | acyl-CoA dehydrogenase | 7.07 | 4.58E-06 |
| *fadA* ^a^ | acetyl-CoA acetyltransferase | 4.70 | 7.11E-06 |
| *fadN* ^a^ | enoyl-CoA hydratase | 4.07 | 5.06E-06 |
| *iolT* ^a^ | myo-inositol transporter | 3.81 | 1.12E-08 |
| *ythQ* | ABC transporter permease | 3.46 | 3.27E-07 |
| *fadF* | iron-sulfur-binding reductase | 3.13 | 1.83E-06 |
| *acdA* ^a^ | acyl-CoA dehydrogenase | 3.03 | 5.40E-06 |
| *fadH* ^a^ | short chain dehydrogenase | 2.96 | 3.46E-06 |
| *gltA* | glutamate synthase large subunit | 2.95 | 2.78E-08 |
| *ythP* | ABC transporter ATP-binding protein | 2.80 | 1.11E-05 |
| *fbpB* | hypothetical protein | 2.67 | 5.71E-06 |
| *lcfB* ^a^ | long-chain-fatty-acid--CoA ligase | 2.62 | 2.40E-05 |
| *gltB* | glutamate synthase subunit beta | 2.60 | 9.11E-08 |
| *ydjP* | peroxydase | 2.60 | 3.80E-06 |
| *yfhM* | hydrolase | 2.53 | 8.53E-06 |
| *dhbC* ^a^ | isochorismate synthase DhbC | 2.49 | 2.40E-05 |
| *ykuN* | flavodoxin | 2.42 | 2.47E-05 |
| *ycnJ* | copper import protein | 2.39 | 4.88E-06 |
| *etfB* ^a^ | electron transfer flavoprotein subunit beta | 2.37 | 3.53E-05 |
| *yyaQ* | hypothetical protein | 2.29 | 3.16E-02 |
| *ysdB* | hypothetical protein | 2.28 | 3.29E-06 |
| *yflN* | metal-dependent hydrolase | 2.26 | 8.18E-04 |
| *spoVAD* | stage V sporulation protein AD | 2.24 | 4.18E-04 |
| *ywjE* | cardiolipin synthetase | 2.22 | 6.17E-03 |
| *citM* | Mg(2+)/citrate complex secondary transporter | 2.20 | 1.53E-03 |
| *ydjO* | hypothetical protein | 2.20 | 2.86E-05 |
| *ycnI* ^a^ | hypothetical protein | 2.20 | 5.08E-05 |
| *bkdB* | branched-chain alpha-keto acid dehydrogenase E2 subunit | 2.18 | 2.71E-06 |
| *ureC* ^a^ | urease subunit alpha | 2.17 | 7.14E-05 |
| *yrhB* | cystathionine gamma-lyase | 2.17 | 5.90E-03 |
| *yorD* ^a^ | hypothetical protein | 2.14 | 6.66E-04 |
| *rpoE* | DNA-directed RNA polymerase subunit delta | 2.13 | 9.64E-05 |
| *ycnK* | DeoR family transcriptional regulator | 2.12 | 1.26E-04 |
| *yhzC* | hypothetical protein | 2.10 | 2.53E-05 |
| *yuaI* | acetyl-transferase | 2.10 | 3.40E-06 |
| *etfA* ^a^ | electron transfer flavoprotein subunit alpha | 2.10 | 2.90E-05 |
| *cydA* | cytochrome bd ubiquinol oxidase subunit I | 2.10 | 6.11E-05 |
| *ureA* | urease subunit gamma | 2.08 | 3.09E-05 |
| *dnaX* | DNA polymerase III subunits gamma and tau | 2.08 | 8.85E-05 |
| *dppD* | dipeptide ABC transporter ATP-binding protein | 2.08 | 1.85E-05 |
| *fadG* ^a^ | Putative peptidoglycan-binding domain-containing protein | 2.06 | 1.67E-05 |
| *srfAD* | surfactin synthetase | 2.05 | 3.42E-04 |
| *dppE* | dipeptide ABC transporter substrate-binding protein | 2.05 | 6.97E-06 |
| *yurN* ^a^ | fructose-amino acid permease | 2.03 | 7.97E-05 |
| *dppC* | dipeptide ABC transporter permease | 2.02 | 3.37E-05 |
| *yncC* | sugar transporter | 2.02 | 6.12E-05 |
| *dppB* | dipeptide ABC transporter permease | 2.01 | 2.43E-05 |
| *yorC* | hypothetical protein | 2.00 | 1.89E-03 |

^a^ also up-regulated under sporulation conditions
